# When GPVI Goes Rogue: Pathogenesis and Therapeutic Horizons in ITP

**DOI:** 10.1017/erm.2026.10047

**Published:** 2026-04-13

**Authors:** Jalal Naghinezhad, Ahmad Mohajerian, Shana Ahadi, Nastaran Khodakarim, Michael R. Hamblin, Hadi Rezaeeyan

**Affiliations:** 1 Department of Medical Laboratory Sciences, School of Allied Medical Science, Mazandaran University of Medical Sciences, Sari, Iran; 2 Department of Emergency Medicine, Faculty of Medicine, Ahvaz Jundishapur University of Medical Sciences, Ahvaz, Iran; 3 School of Medicine, Jundishapur University of Medical Sciences, Ahvaz, Iran; 4 Department of Internal Medicine, School of Medicine, Hazrat-e Rasool General Hospital, Iran University of Medical Sciences, Tehran, Iran; 5 Laser Research Centre, University of Johannesburg, Doornfontein, South Africa; 6 Asadabad School of Medical Sciences, Asadabad, Iran

**Keywords:** drug delivery systems, glycoprotein VI, hitchhiking, hydrogel, immune thrombocytopenia, nanoparticle, platelet activation

## Abstract

Immune thrombocytopenia (ITP) is a heterogeneous autoimmune disorder characterized by immune-mediated platelet destruction, impaired thrombopoiesis and a bleeding diathesis, with growing recognition of accompanying inflammatory and immunoregulatory disturbances. Despite the widespread use of corticosteroids, intravenous immunoglobulin (IVIG) and thrombopoietin receptor agonists (TPO-RAs), 30–50% of patients exhibit incomplete, unstable or transient platelet responses, underscoring a persistent unmet need for mechanism-informed therapeutic strategies. Current management paradigms remain largely focused on platelet count restoration rather than direct modulation of pathogenic platelet–immune crosstalk. Accumulating pre-clinical evidence, supported by limited clinical observations, implicates platelet glycoprotein VI (GPVI), a collagen receptor and immunothrombotic signalling hub, as a mechanistically relevant contributor to platelet hyperactivation, inflammatory amplification and aberrant platelet–leucocyte interactions in ITP. Experimental models indicate that GPVI-dependent signalling pathways can promote thromboinflammatory responses, facilitate immune cell engagement and influence platelet clearance dynamics, positioning GPVI as a plausible, albeit incompletely validated, therapeutic target. Emerging pre-clinical studies suggest that selective modulation of GPVI signalling may attenuate pathogenic platelet activation while preserving essential haemostatic function, thereby improving platelet survival and functional competence. This review integrates current insights into GPVI biology within the broader immunopathological landscape of ITP and evaluates innovative therapeutic concepts, including GPVI-targeted inhibitors deployed through nanocarrier systems, autologous platelet-mediated delivery and hydrogel-based protective platforms designed to enhance targeting precision and durability. We further discuss the rationale for combination strategies with established therapies and the potential utility of GPVI-linked biomarkers and platelet functional profiling to guide patient stratification. By reframing platelets as active immunoregulatory effectors rather than passive autoimmune targets, this review advances a mechanistic framework for next-generation, precision-oriented intervention in ITP. Although clinical validation remains limited, GPVI-centred strategies represent a rational and testable avenue for moving beyond symptomatic platelet augmentation towards disease-modifying immunothrombotic modulation.

## Graphical abstract

**Figure:**
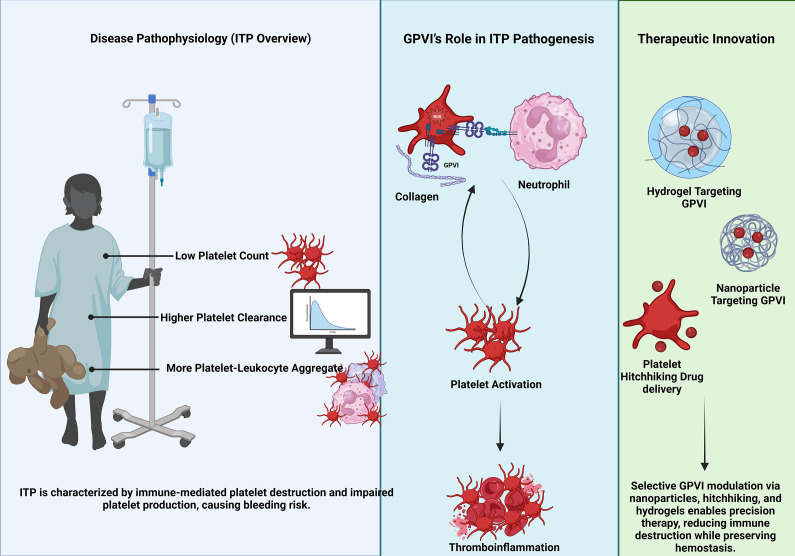

## Highlights

1. GPVI emerges as a mechanistically relevant contributor to platelet hyperactivation and immune dysregulation in ITP, supported primarily by pre-clinical and limited clinical evidence.
2. Current ITP therapies remain sub-optimal, with 30–50% of patients showing incomplete or transient platelet recovery, underscoring the need for mechanism-informed therapeutic strategies.
3. GPVI-targeted approaches hold potential for selectively modulating pathogenic platelet activation while preserving essential haemostatic function.
4. Advanced delivery platforms, including nanoparticles, platelet hitchhiking and protective hydrogel systems, may enhance targeting precision and reduce systemic toxicity.
5. Rational combination of GPVI modulation with standard therapies (TPO-RAs, IVIG, corticosteroids) represents a testable strategy to improve platelet survival and functional recovery.
6. Patient stratification using GPVI-related biomarkers and platelet functional assays may enable more personalized therapeutic decision making.
7. Targeting platelet–immune crosstalk through GPVI signalling pathways represents a conceptual shift towards mechanism-driven and precision-oriented ITP management.

## Introduction

### Clinical overview of ITP

Immune thrombocytopenia (ITP) is a heterogeneous autoimmune disorder characterized by reduced platelet counts and increased bleeding risk, resulting from a combination of immune-mediated platelet destruction and impaired thrombopoiesis (Ref. 1). Clinically, ITP exhibits wide phenotypic diversity, ranging from asymptomatic thrombocytopenia to severe, life-threatening haemorrhage, including intracranial bleeding (Ref. 2). This spectrum of presentations underscores the variable pathogenic mechanisms at play and the challenges in predicting disease course. Insights from related platelet disorders, such as thrombotic thrombocytopenic purpura (TTP), further highlight the clinical complexity of platelet-mediated diseases: TTP demonstrates diverse manifestations including fluctuating neurological symptoms, fever, renal impairment and variable thrombocytopenia, influenced by endothelial activation, autoantibodies and platelet aggregation factors (Ref. 3). Although ITP differs mechanistically from TTP, this comparison emphasizes the broader principle that platelet disorders can present with highly heterogeneous clinical phenotypes.

### Unmet needs in ITP management

Despite advances in therapeutic options, current ITP management remains largely reactive, focusing on symptom control rather than addressing the underlying pathogenic mechanisms (Ref. 4). Many patients exhibit persistent platelet hyperactivation, immune dysregulation or thromboinflammatory complications despite conventional therapy (Ref. 1). Moreover, broad immunosuppressive strategies carry significant side effects, including infection risk, metabolic complications and bone marrow suppression (Refs 5–7). These limitations underscore the urgent need for precision-guided approaches that can selectively modulate pathogenic pathways while preserving essential haemostasis, ultimately improving both clinical outcomes and patient quality of life.

### Platelet receptors beyond count: the emerging role of GPVI

Recent research has shifted attention from platelet quantity alone towards qualitative dysfunction and receptor-mediated pathogenic mechanisms in ITP (Ref. 8). Glycoprotein VI (GPVI), a platelet-specific collagen receptor, has emerged as a central mediator linking platelet hyperactivation to immune dysregulation (Ref. 9). GPVI drives thromboinflammatory signalling, promotes platelet–leucocyte crosstalk and contributes to accelerated platelet clearance, placing it at the nexus of ITP pathophysiology (Refs 10, 11). Its selective expression on platelets and megakaryocytes offers a unique therapeutic opportunity: targeted modulation of GPVI could reduce immune-mediated destruction, limit pathogenic platelet activation and preserve essential haemostatic function (Refs 12, 13).

Current therapies achieve variable response rates, with roughly 30–50% of patients showing incomplete or transient platelet recovery despite corticosteroids, IVIG or TPO receptor agonists, highlighting the need for more mechanistically precise interventions (Ref. 14). By moving beyond symptom control towards targeted, biology-driven therapy, GPVI modulation introduces the promise of precision medicine in ITP. This article will explore GPVI biology, its pathogenic role in ITP and innovative therapeutic strategies, including nanoparticle delivery, platelet hitchhiking and protective hydrogel systems, providing a framework for next-generation, patient-tailored treatment approaches.

## GPVI biology and platelet function

### Structure and signalling pathways of GPVI

GPVI is a type I transmembrane immunoglobulin-like receptor that is expressed almost exclusively on platelets and megakaryocytes (Ref. 15). As the primary collagen receptor on the platelet surface, GPVI plays a pivotal role in initiating platelet adhesion and activation at sites of vascular injury (Ref. 16). Structurally, GPVI is organized as a dimer on the platelet membrane, a configuration that is critical for high-affinity ligand binding and efficient signal transduction (Refs 17, 18). Its functional activity relies on a non-covalent association with the Fc receptor γ-chain (FcRγ), which contains an immunoreceptor tyrosine-based activation motif (ITAM) (Ref. 19). This ITAM is indispensable for propagating intracellular signalling upon receptor engagement.

Upon exposure to its canonical ligands – primarily sub-endothelial collagen, but also laminin and fibrin – the extracellular domains of GPVI cluster, bringing associated FcRγ chains into close proximity (Ref. 20). This clustering triggers phosphorylation of ITAM tyrosines by Src family kinases, including Lyn and Fyn, which serve as proximal initiators of the signalling cascade (Ref. 21). The phosphorylated ITAMs then recruit spleen tyrosine kinase (Syk), a critical mediator that amplifies and propagates downstream signalling (Ref. 22). Syk activation engages adaptor proteins such as linker for activation of T cells (LAT) and SH2 domain-containing leucocyte protein of 76 kDa (SLP-76), forming a multi-protein signalling complex at the platelet membrane (Ref. 23). This complex activates phospholipase C gamma 2 (PLCγ2), leading to the hydrolysis of phosphatidylinositol 4,5-bisphosphate (PIP2) into diacylglycerol (DAG) and inositol 1,4,5-trisphosphate (IP3) (Ref. 24).

IP3-mediated release of calcium from intracellular stores, along with DAG-driven protein kinase C (PKC) activation, orchestrates a wide array of platelet responses (Ref. 25). These include shape change, granule secretion of ADP, thromboxane A2 and pro-inflammatory mediators, and the inside-out activation of integrin αIIbβ3, which facilitates fibrinogen binding and platelet aggregation (Ref. 26). This cascade underscores GPVI’s role as a master regulator of platelet reactivity, linking mechanical cues from the extracellular matrix to potent intracellular signalling networks.

Beyond haemostasis, GPVI signalling intersects with inflammatory and immune pathways (Ref. 27). Activated platelets can release cytokines and chemokines, form platelet–leucocyte aggregates and influence endothelial function, highlighting GPVI as a critical node at the interface of thrombosis and inflammation (Ref. 28). Dysregulation of this pathway, as observed in ITP and other platelet-mediated disorders, not only impacts platelet survival and function but may also contribute to aberrant immune activation and vascular complications (Ref. 1).

**Figure 1.**
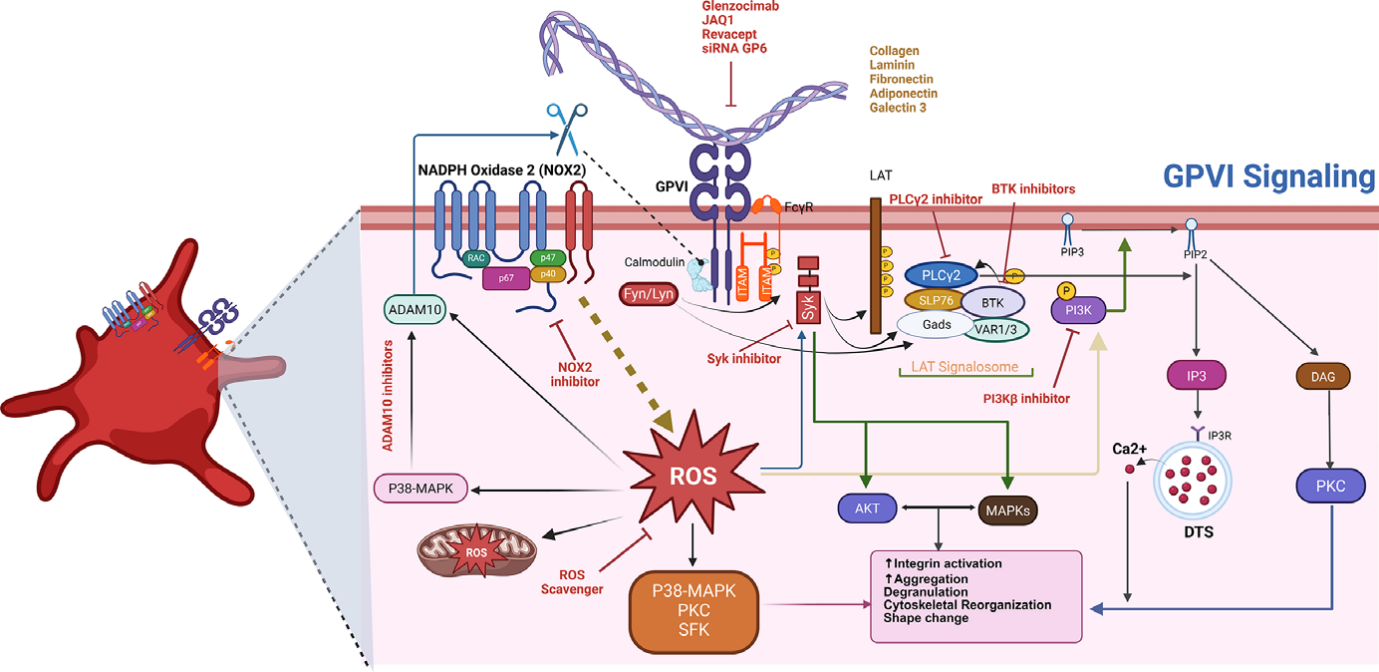
Therapeutic intervention points in the GPVI signalling pathway. Glycoprotein VI (GPVI) is a key platelet receptor that detects components of the exposed vascular matrix – such as fibrillar collagen, fibrin, fibronectin, galectin-3, laminin and adiponectin – following vascular injury. Engagement of these ligands prompts the clustering of GPVI in association with its signalling partner, FcRγ, initiating a cascade of intracellular events. This receptor clustering activates Src family kinases, which phosphorylate immunoreceptor tyrosine-based activation motifs (ITAMs) within FcRγ. These phosphorylated motifs recruit and activate the kinase Syk, leading to the assembly of a downstream signalling hub centred around the adaptor proteins LAT and SLP-76. This signalosome subsequently activates phospholipase C gamma 2 (PLCγ2), which catalyses the hydrolysis of PIP₂ into two second messengers – DAG and IP₃. These signalling molecules promote calcium release from intracellular stores and protein kinase C (PKC) activation, collectively driving platelet shape change, granule secretion and integrin activation. In parallel, PI3Kβ is activated and contributes to signal amplification through the Akt pathway, enhancing the activation of integrins α2β1 and αIIbβ3 and supporting sustained aggregation. Additional regulatory inputs include the generation of reactive oxygen species (ROS) via NADPH oxidase complexes, primarily NOX2, which reinforce kinase signalling. Furthermore, GPVI availability at the platelet surface is modulated by proteolytic cleavage through metalloproteinases – primarily ADAM10, with context-specific involvement of ADAM17.

In summary, GPVI integrates structural precision, ligand specificity and complex intracellular signalling to control platelet activation, adhesion and crosstalk with the immune system, positioning it as both a mechanistic cornerstone of platelet biology and a compelling therapeutic target (see Figure 1).

### GPVI in platelet activation and adhesion

GPVI functions as a primary receptor for sub-endothelial collagen and related extracellular matrix ligands (e.g. laminin, fibrin), rapidly initiating platelet spreading, actin reorganization and integrin recruitment that secure firm adhesion at sites of vascular injury (Refs 29, 30). Downstream GPVI signalling triggers granule secretion (ADP, thromboxane A₂), amplifies inside-out activation of integrin αIIbβ3 and promotes platelet recruitment across a range of shear conditions, thereby consolidating thrombus formation while restricting systemic activation (Refs 30–32).

Beyond classical haemostasis, GPVI-dependent activation exerts important immunomodulatory effects. Activated platelets engage with leucocytes to form platelet–neutrophil, platelet–monocyte and platelet–lymphocyte aggregates, which potentiate cytokine and chemokine release, augment immune cell recruitment and modulate local inflammatory responses (Ref. 33). In immune-mediated thrombocytopenias such as ITP, aberrant GPVI signalling may therefore contribute to pathological platelet clearance and vascular inflammation by exposing pro-phagocytic signals and enhancing platelet–immune cell crosstalk.

Importantly, autoantibodies targeting platelets are not only unique to primary, organ-restricted diseases but also occur in systemic, non-organ-specific autoimmune disorders. In systemic lupus erythematosus (SLE), for example, platelet-associated and circulating anti-platelet antibodies have been documented repeatedly and are implicated in SLE-associated thrombocytopenia (Refs 34, 35). Moreover, systemic inflammatory mediators – including chemokines such as monocyte chemoattractant protein-1 (MCP-1) and other cytokines – correlate with disease activity and haematological abnormalities in SLE and can contribute to reduced platelet counts via immune-mediated mechanisms (Refs 36–38).

Additionally, platelet counts and function can be altered in patients with malignancies. Tumours secrete a variety of cytokines and growth factors, including interleukins, tumour necrosis factor-alpha and vascular endothelial growth factor, which can modulate platelet activation, adhesion and aggregation through up-regulation of adhesive molecules on platelet surfaces (Refs 39–41). These tumour-derived mediators can induce systemic thromboinflammatory states, resulting in accelerated platelet clearance and reduced circulating platelet counts. In some cases, this process resembles immune-mediated thrombocytopenia, where cytokine-driven platelet activation and adhesion contribute to both qualitative platelet dysfunction and quantitative thrombocytopenia, akin to mechanisms observed in ITP.

Taken together, these data position GPVI at the intersection of platelet adhesion, thrombus consolidation and immune-thromboinflammatory signalling. This dual role underlies the rationale for investigating GPVI-targeted interventions in ITP and related immune-mediated platelet disorders, while also underscoring the need to account for systemic autoimmune mechanisms (antibody- and cytokine-driven) when interpreting platelet phenotypes and designing translational studies.

### Crosstalk between GPVI and other platelet receptors (e.g. αIIbβ3, CLEC-2)

GPVI signalling operates within a highly interconnected network of platelet surface receptors, integrating multiple inputs to fine-tune platelet activation, aggregation and thromboinflammatory responses (Ref. 42). One of the most critical interactions is with the integrin αIIbβ3, the major fibrinogen receptor on platelets. Collagen engagement of GPVI primes αIIbβ3 through inside-out signalling, increasing its affinity for fibrinogen and von Willebrand factor, thereby enhancing platelet aggregation and stabilizing thrombus formation under shear stress (Ref. 43). This crosstalk ensures that adhesion at sites of vascular injury rapidly transitions into a firm, cohesive platelet plug (Ref. 44).

GPVI also interacts functionally with C-type lectin-like receptor 2 (CLEC-2), a receptor involved in platelet–immune cell communication and maintenance of vascular integrity (Ref. 45). Cooperative signalling between GPVI and CLEC-2 modulates thromboinflammatory pathways, including the formation of platelet–leucocyte aggregates and release of pro-inflammatory cytokines, linking haemostasis with immune regulation (Ref. 46). Beyond αIIbβ3 and CLEC-2, GPVI communicates with other ITAM-linked receptors and non-ITAM receptors, creating a layered network that calibrates platelet sensitivity to vascular injury versus systemic activation (Ref. 47).

This receptor crosstalk allows platelets to integrate environmental cues, balancing the need for rapid haemostasis against the risk of excessive thrombosis or immune-mediated vascular damage (Ref. 48). In the context of ITP and other pathological states, dysregulation of these interactions may amplify platelet activation, promote immune-mediated clearance and exacerbate thromboinflammatory complications (Ref. 1). Understanding the interdependence of GPVI with αIIbβ3, CLEC-2 and other receptors provides a mechanistic framework to identify therapeutic windows where GPVI-targeted interventions can selectively suppress pathological platelet activation while preserving essential haemostatic function.

## GPVI in ITP pathogenesis

### GPVI expression in ITP: evidence from clinical and pre-clinical studies

Accumulating evidence indicates that GPVI expression and function are dysregulated in ITP, contributing not only to quantitative platelet loss, but also to functional impairment and immune dysregulation (Ref. 49). Clinical investigations reveal heterogeneous GPVI expression profiles among ITP patients (Refs 50, 51). Limited clinical evidence, including individual case reports, suggests that circulating platelets may exhibit reduced GPVI surface density in ITP, potentially due to autoantibody-mediated receptor shedding or internalization (Ref. 52). Shedding of GPVI is mediated by metalloproteinases such as ADAM10, which are up-regulated in response to immune complexes and inflammatory cytokines, resulting in loss of collagen-binding capacity and impaired platelet activation (Ref. 53). Conversely, some patient sub-groups demonstrate compensatory up-regulation of GPVI on remaining platelets, suggesting a dynamic adaptive response aimed at preserving haemostatic function despite thrombocytopenia (Ref. 54).

Pre-clinical models corroborate these clinical observations. Murine ITP models using anti-platelet antibodies demonstrate that GPVI is a direct target for pathogenic autoantibodies, triggering aberrant signalling cascades even in the absence of overt thrombocytopenia (Refs 55, 56). This GPVI engagement can potentiate platelet activation, promote granule release and increase the susceptibility of platelets to phagocytic clearance (Ref. 26). Indirect mechanisms have also been described, where autoantibody binding to other platelet surface antigens leads to crosslinking and secondary GPVI activation or shedding, highlighting the receptor’s centrality in ITP pathophysiology.

Importantly, these findings establish GPVI as more than a passive marker of platelet count or activation; it actively shapes disease dynamics. Altered GPVI expression and signalling contribute to a feed-forward loop in which platelet hyperactivation, immune complex formation and clearance converge, amplifying both thrombocytopenia and systemic immune dysregulation. The clinical and mechanistic insights underscore GPVI’s dual role in mediating haemostatic dysfunction and immune perturbation in ITP, positioning it as a prime candidate for targeted therapeutic strategies aimed at modulating platelet survival, activation and immune crosstalk.

### GPVI-mediated platelet activation and immune dysregulation

GPVI signalling extends far beyond its classical role in haemostasis, emerging as a central mediator of immune modulation in ITP. Engagement of GPVI by autoantibodies, exposed collagen or other extracellular matrix ligands triggers robust platelet activation characterized by cytoskeletal rearrangement, granule secretion and up-regulation of adhesion molecules such as P-selectin and CD40L (Ref. 57). These surface molecules enable platelets to directly interact with leucocytes, including T cells, B cells and monocytes, thereby influencing adaptive and innate immune responses (Refs 58, 59).

Hyperactivated GPVI signalling fosters a pro-inflammatory environment in ITP. Platelets release a spectrum of cytokines, chemokines and extracellular vesicles, including IL-1β, CD40L-containing microparticles and platelet factor 4 (PF4), which can enhance dendritic cell maturation, skew T-helper cell polarization towards pro-autoimmune phenotypes and promote B-cell autoantibody production (Ref. 60). This creates a feed-forward loop in which platelet hyperactivation amplifies immune-mediated destruction, perpetuating thrombocytopenia and systemic inflammation (Ref. 61).

Moreover, GPVI-mediated platelet activation contributes to aberrant platelet–leucocyte aggregates, which facilitate immune complex deposition and enhance Fc receptor–mediated phagocytosis of platelets by splenic macrophages (Ref. 62). This mechanistic axis underscores how dysregulated GPVI signalling links platelet hyperactivation to immune dysregulation, positioning GPVI at the interface of thrombosis, inflammation and autoimmunity. Consequently, targeting GPVI may not only modulate platelet activation but also attenuate maladaptive immune responses, offering a dual therapeutic benefit in ITP.

### Role of GPVI in platelet destruction and clearance

GPVI plays a pivotal role in determining the fate of platelets in ITP, linking receptor activation to accelerated platelet clearance (Refs 63, 64). Hyperactivation of GPVI exposes ‘eat-me’ signals on the platelet surface, most notably phosphatidylserine, which serves as a recognition cue for splenic macrophages and dendritic cells (Ref. 64). This exposure enhances Fc receptor–mediated phagocytosis, effectively reducing circulating platelet numbers.

Autoantibody interactions with GPVI further exacerbate platelet vulnerability. Binding of anti-platelet autoantibodies can trigger receptor shedding, internalization or conformational changes that compromise platelet structural integrity (Refs 65, 66). These processes not only diminish functional GPVI signalling but also mark platelets for premature clearance by the reticuloendothelial system.

Additionally, GPVI-driven platelet activation can promote the formation of platelet microparticles and immune complexes, which further recruit phagocytic cells and propagate systemic inflammation (Ref. 67). This establishes a feed-forward loop where GPVI-mediated hyperactivation accelerates both functional impairment and quantitative loss of platelets. Collectively, these mechanisms underscore GPVI as a central mediator of platelet destruction in ITP, linking receptor-level dysregulation to both thrombocytopenia and disease progression, and highlighting its potential as a therapeutic target.

### GPVI and platelet–leucocyte interactions in ITP

Accumulating experimental evidence suggests that GPVI-mediated platelet activation contributes to pathogenic platelet–leucocyte crosstalk in ITP, primarily as an amplifying immunothrombotic mechanism rather than a singular initiating driver of disease (Ref. 26). Upon activation, platelets expressing GPVI engage in dynamic interactions with circulating leucocytes – including monocytes and neutrophils, and to a lesser extent lymphocytes – forming platelet–leucocyte aggregates that have been predominantly characterized in pre-clinical and ex vivo human systems (Refs 68, 69).

Within these aggregates, activated platelets facilitate localized immune activation through the release of pro-inflammatory mediators (e.g. IL-1), chemokines (e.g. PF4, RANTES) and reactive oxygen species, thereby shaping leucocyte effector function and sustaining inflammatory signalling (Refs 70, 71). Experimental studies further suggest that platelet–leucocyte interactions can enhance antigen-presentation capacity and modulate innate immune responses, processes that may indirectly support autoantibody production and chronic immune activation in ITP (Ref. 72). Complement activation within platelet–leucocyte complexes has also been observed in experimental settings, potentially increasing platelet opsonization and susceptibility to clearance (Ref. 54).

Importantly, GPVI appears to function as a molecular interface linking platelet activation to immune cell engagement, operating within a broader pathogenic network that includes Fcγ-receptor–mediated platelet clearance, cytokine-driven immune dysregulation and splenic macrophage-dependent platelet destruction (Ref. 26). While emerging clinical observations – including limited case-based human data – support the biological plausibility of GPVI involvement, robust cohort-level evidence demonstrating sustained platelet recovery through GPVI modulation in ITP remains limited (Ref. 73).

Nevertheless, by positioning GPVI as a context-dependent immunothrombotic amplifier, rather than a dominant pathogenic determinant, therapeutic strategies targeting GPVI-mediated platelet–leucocyte crosstalk may hold value as adjunctive or precision-based interventions. Such approaches could potentially attenuate immune-mediated platelet destruction and inflammatory amplification when integrated with established immunomodulatory therapies (Table 1).

**Table 1.** GPVI biology, signalling and its role in ITP pathogenesis

Domain	Key concepts	Mechanistic details	Functional consequences	Pathological implications in ITP
Structure and expression	GPVI is a platelet-specific, type I transmembrane immunoglobulin-like receptor	Exists as a dimer; non-covalently associates with FcRγ (ITAM-containing)	Enables high-affinity collagen binding and robust signal transduction	GPVI expression is reduced or dysregulated via shedding/internalization triggered by autoantibodies
Ligands and activation	Binds sub-endothelial collagen, fibrin and laminin	Ligand binding induces GPVI clustering → FcRγ ITAM phosphorylation by Src kinases (Lyn, Fyn)	Initiates platelet activation at injury sites	Autoantibodies or immune complexes aberrantly activate GPVI even in absence of injury
Signal transduction	Activates downstream Syk, LAT, SLP-76 and PLCγ2	PIP2 hydrolysis → DAG + IP3 → Ca^2+^ mobilization + PKC activation	Triggers shape change, granule release (ADP, thromboxane A2) and integrin αIIbβ3 activation	Hyperactivation promotes platelet aggregation, microparticle release and inflammation
Platelet function	Coordinates adhesion, spreading and thrombus formation	Actin re-modelling, integrin recruitment (filopodia, lamellipodia)	Stabilizes platelet adhesion under flow; ensures clot integrity	Dysregulated GPVI signalling leads to dysfunctional clotting or excessive thromboinflammation
Receptor crosstalk	Interacts with αIIbβ3, CLEC-2 and other receptors	Inside-out signalling enhances αIIbβ3 affinity; CLEC-2 cooperation modulates immune responses	Integrates vascular injury signals with immune cell interaction	Crosstalk amplifies immune-mediated platelet clearance and vascular inflammation in ITP
Inflammatory Interface	Platelets serve immune functions via GPVI	Up-regulation of CD40L, P-selectin; secretion of IL-1β, PF4, chemokines	Platelet–leucocyte aggregates form; enhance antigen presentation and cytokine storms	Drives systemic immune activation, autoantibody production and complement activation in ITP
Platelet clearance mechanisms	GPVI hyperactivation exposes ‘eat-me’ signals (e.g. phosphatidylserine)	Promotes FcγR-mediated phagocytosis by macrophages; triggers receptor shedding by ADAM10	Leads to platelet removal via splenic and hepatic clearance pathways	Accelerates thrombocytopenia; creates a feed-forward cycle of destruction and immune stimulation
Immune dysregulation	Platelets modulate innate and			

## GPVI as a therapeutic target in ITP

### Rationale for targeting GPVI

GPVI represents a highly selective and mechanistically compelling therapeutic target in ITP, given its central role in both platelet activation and immune modulation. Unlike conventional anti-platelet or immunosuppressive therapies, which broadly impair haemostasis or immunity, GPVI is predominantly expressed on platelets and megakaryocytes, allowing targeted intervention with minimal off-target effects (Ref. 12). In ITP, aberrant GPVI signalling drives excessive platelet activation, exposes pro-phagocytic signals and promotes the formation of platelet–leucocyte aggregates, creating a feed-forward loop that amplifies immune-mediated platelet destruction (Ref. 56).

By specifically modulating GPVI, it is possible to dampen pathological platelet hyperactivity while preserving the function of other platelet receptors essential for normal haemostasis (Ref. 10). This dual effect – reducing both immune-driven clearance and platelet-mediated inflammatory signalling – addresses the core pathogenic mechanisms of ITP rather than merely mitigating symptoms. Moreover, GPVI-targeted therapy offers the potential to synergize with existing treatments, complementing platelet production and immune suppression with protection against ongoing platelet destruction.

Overall, GPVI inhibition provides a precision-based, mechanistically grounded approach: it intervenes at the intersection of thrombosis and immunity, directly targeting the pathways that link platelet hyperactivation to immune dysregulation in ITP, thereby offering a pathway towards both functional and quantitative restoration of platelet homeostasis.

### Pre-clinical evidence for GPVI inhibitors in thrombocytopenic conditions

Robust pre-clinical data support GPVI inhibition as a promising therapeutic strategy in thrombocytopenic conditions, including ITP (Ref. 74). In murine models, anti-GPVI monoclonal antibodies, soluble GPVI-Fc decoys and small-molecule inhibitors consistently reduce pathological platelet activation while sparing essential haemostatic function (Refs 75, 76). These interventions diminish platelet–leucocyte aggregate formation, lower exposure of pro-phagocytic signals and attenuate autoimmune-mediated platelet clearance, directly addressing core mechanisms driving thrombocytopenia.

Beyond platelet preservation, GPVI blockade modulates systemic immune responses, reducing pro-inflammatory cytokine release and tempering the feed-forward loop of immune dysregulation that exacerbates ITP (Ref. 12). Importantly, these studies demonstrate that targeted inhibition of GPVI can decouple pathogenic platelet hyperactivity from normal haemostasis – a critical consideration for safety in clinical translation (Ref. 77). Collectively, pre-clinical evidence establishes GPVI as a dual-function target, capable of mitigating both quantitative platelet loss and qualitative platelet-mediated immune dysfunction, providing a strong mechanistic rationale for clinical evaluation in ITP.

### Potential benefits: reducing platelet activation and immune-mediated destruction

Targeting GPVI in ITP offers a mechanistically coherent strategy to concurrently modulate platelet hyperactivation and platelet–immune crosstalk, two inter-linked processes implicated in immune-mediated platelet loss (Ref. 12). In experimental systems, inhibition of GPVI attenuates aberrant platelet activation, limits the exposure of pro-phagocytic and pro-inflammatory platelet signals and reduces platelet–leucocyte aggregate formation, thereby dampening downstream immune amplification pathways, including cytokine release, complement engagement and sustained inflammatory signalling (Refs 1, 12, 74). These effects position GPVI at the interface of thrombosis and immunity, providing a rational molecular node for intervention.

GPVI modulation may also complement established ITP therapies, including TPO receptor agonists, IVIG and corticosteroids, by protecting newly generated platelets from immune-mediated clearance while allowing restoration of platelet mass through enhanced thrombopoiesis. In this framework, GPVI-directed strategies are not envisioned as replacements for current standards of care but as adjunctive, mechanism-informed approaches with the potential to improve the durability of platelet responses and stabilize haemostatic function in selected patient subsets.

Recent pre-clinical advances further underscore the therapeutic tractability of GPVI. The humanized anti-GPVI Fab EMA601 exemplifies next-generation GPVI inhibitors with markedly enhanced affinity for human GPVI (reported K_D ≈ 0.195 nM) and superior in vitro potency compared with earlier GPVI-targeting constructs (Ref. 78). In hGPVI-expressing murine models, EMA601 effectively suppressed GPVI-dependent platelet activation and conferred protection in experimental arterial thrombosis and ischaemic stroke paradigms, without prolongation of bleeding times in the assays employed (Ref. 79). Although these findings provide important proof of principle for high-affinity GPVI blockade, the evidence base remains pre-clinical and no clinical evaluation of EMA601 in ITP or other immune-mediated thrombocytopenias has yet been reported. Accordingly, EMA601 illustrates both the translational potential of GPVI-directed inhibition and the substantial evidentiary gap that must be bridged before such strategies can be credibly advanced towards clinical application in ITP.

### Safety considerations: bleeding risk and immune implications

Although GPVI presents a highly promising therapeutic target in ITP, safety remains paramount. Human studies indicate that complete GPVI deficiency produces only mild bleeding tendencies, suggesting that partial or carefully titrated inhibition can effectively limit pathological platelet activation while preserving essential haemostatic function (Refs 80, 81).

Beyond bleeding risk, GPVI-targeted therapies must be evaluated for potential immunological consequences. Platelets play key roles in host defence and inflammatory regulation, and GPVI modulation could theoretically alter pathogen recognition or platelet-driven immune signalling (see Table 2). However, pre-clinical and early clinical studies demonstrate that selective GPVI blockade can achieve a favourable therapeutic window, suppressing immune-mediated platelet destruction and thromboinflammatory activation without compromising global haemostasis. These findings support the feasibility of safely integrating GPVI-targeted interventions into ITP management while minimizing adverse effects.

**Table 2.** Therapeutic targeting of GPVI in immune thrombocytopenia: mechanistic rationale, pre-clinical validation and clinical translation

Domain	Focus	Scientific insights and therapeutic implications
Mechanistic rationale	Selective expression	GPVI is restricted to platelets and megakaryocytes, allowing targeted intervention without broad immunosuppression or haemostatic compromise.
	Pathogenic role in ITP	Aberrant GPVI signalling promotes platelet activation, pro-phagocytic signalling and platelet–leucocyte aggregates, establishing a feed-forward loop of immune-mediated destruction.
	Therapeutic target profile	Modulation of GPVI uncouples pathological platelet activity from essential haemostatic function, addressing both immune dysregulation and thrombocytopenia.
Pre-clinical validation	Efficacy in animal models	Anti-GPVI monoclonal antibodies, GPVI-Fc fusion proteins and small-molecule inhibitors reduce platelet–leucocyte aggregates, opsonization and phagocytosis in murine ITP models.
	Immune modulatory effects	GPVI inhibition attenuates systemic inflammatory signalling and cytokine release, mitigating the immune amplification loop characteristic of chronic ITP.
	Safety and haemostasis	Targeted GPVI blockade preserves primary haemostasis in vivo, with no significant increase in bleeding in models of thrombocytopenia.
Therapeutic potential	Dual-mechanism intervention	Reduces both quantitative platelet loss and qualitative immune-mediated dysfunction.
	Synergism with standard therapies	Enhances efficacy of TPO receptor agonists by protecting nascent platelets; allows dose-sparing of corticosteroids and IVIG.
	Towards disease modification	Enables sustained platelet recovery and reduced inflammatory burden, offering potential for durable remission beyond symptomatic control.
Safety considerations	Haemostatic reserve	Congenital GPVI deficiency in humans is associated with only mild bleeding, supporting a favourable safety profile for partial pharmacologic inhibition.
	Immunological integrity	Despite platelets’ roles in innate immunity, selective GPVI modulation shows no significant immunosuppression in pre-clinical studies.
Translational pathway	Patient stratification	Optimal candidates include those with persistent platelet hyperactivation, elevated GPVI-related biomarkers or refractoriness to TPO-RAs and IVIG.
	Candidate biomarkers	Surface GPVI density, soluble GPVI, platelet activation markers (e.g. P-selectin, PAC-1), platelet–leucocyte aggregates and inflammatory cytokine signatures.
	Precision medicine framework	Enables therapy alignment with individual pathogenic profiles, maximizing response and minimizing off-target risk.
Combination strategies	Mechanistic rationale	GPVI inhibitors address a therapeutic gap by mitigating immune clearance and platelet dysfunction concurrently with stimulatory or immunosuppressive agents.
	Clinical application	Rational combinations may enhance platelet count durability, reduce bleeding risk and improve patient-reported outcomes.
Clinical monitoring and biomarker development	Dynamic biomarker utility	Longitudinal monitoring of GPVI activity, platelet–immune interactions and cytokine flux informs therapeutic dosing, response prediction and safety surveillance.
	Integration into trial design	Mechanistically anchored biomarkers enable adaptive clinical trials, enrich responder populations and accelerate pathway-informed drug development.

## Translational and clinical considerations

### Patient stratification: who may benefit from GPVI inhibition?

Precision patient selection is critical for the successful translation of GPVI-targeted therapies in ITP. Not all patients exhibit the same degree of platelet hyperactivation or immune-mediated platelet destruction, and tailoring therapy to the underlying pathogenic profile can maximize benefit while minimizing risk. Ideal candidates for GPVI inhibition are likely to include individuals with persistent platelet hyper-reactivity, elevated levels of platelet–leucocyte aggregates or ongoing autoantibody-mediated platelet clearance despite optimized standard treatments, such as corticosteroids, IVIG or TPO receptor agonists (Ref. 1).

Emerging biomarkers can guide stratification and therapeutic decision making. Surface GPVI expression and shedding, plasma soluble GPVI levels and functional assays assessing platelet responsiveness to collagen or immune stimuli provide quantitative and mechanistic insight into the contribution of GPVI-mediated pathways in individual patients (Refs 82, 83). Coupling these readouts with measures of systemic inflammation – such as circulating cytokines, chemokines and thromboinflammatory markers – can identify patients with a high pathogenic burden, who are most likely to benefit from selective GPVI modulation.

Importantly, patients who are refractory to TPO receptor agonists, or those with high thromboinflammatory activity, may gain particular advantage, as GPVI-targeted interventions simultaneously mitigate pathological platelet activation and preserve residual platelet counts. Early and precise stratification not only enhances the likelihood of therapeutic success but also limits exposure in patients less likely to respond, optimizing both efficacy and safety. By integrating molecular, functional and clinical indicators, patient-specific GPVI profiling may enable a precision-medicine approach in ITP, shifting therapy from generalized immunosuppression towards targeted modulation of pathogenic platelet pathways.

### Combination therapy with standard ITP treatments (e.g. TPO-RA, steroids, IVIG)

GPVI-targeted interventions are unlikely to function as standalone therapies in ITP but are best conceptualized as adjuncts to existing treatment backbones, with the potential to address mechanistically distinct disease components across clinical subtypes of ITP (Refs 84, 85). ITP is increasingly recognized as a heterogeneous disorder encompassing dominant patterns of platelet destruction, impaired platelet production or combined immune–thromboinflammatory phenotypes (Ref. 1). In patients with destruction-predominant disease, characterized by heightened platelet activation, Fc-mediated phagocytosis and platelet–leucocyte aggregation, GPVI inhibition may preferentially attenuate immune recognition and clearance of circulating platelets. In contrast, in patients with production-limited phenotypes, GPVI-directed strategies may be most effective when combined with TPO-RAs, which expand platelet output but do not intrinsically protect nascent platelets from immune-mediated elimination (Refs 86, 87).

Mechanistically, combination therapy can be rationally tailored. TPO-RAs restore platelet mass, while GPVI inhibition may preserve platelet lifespan and limit pathological platelet–immune crosstalk, thereby improving the durability of platelet responses (Ref. 12). Co-administration with IVIG or corticosteroids may further dampen Fc receptor–dependent phagocytosis and systemic immune activation, creating a layered therapeutic strategy that targets both platelet-intrinsic activation pathways and extrinsic immune clearance mechanisms. This framework raises the possibility of dose de-escalation of generalized immunosuppression in selected patients, potentially reducing cumulative steroid exposure and long-term toxicity.

Patient stratification will be essential to the successful clinical translation of such combination approaches. Candidate biomarkers to guide therapeutic selection may include: (i) markers of platelet activation (e.g. surface or soluble P-selectin, platelet-derived extracellular vesicles) (Ref. 88), (ii) indices of platelet–leucocyte interactions or circulating platelet–neutrophil aggregates (Ref. 68), (iii) GPVI expression levels or functional responsiveness in ex vivo platelet activation assays (Ref. 50) and (iv) immunophenotypic features reflecting dominant effector mechanisms (e.g. Fcγ receptor expression profiles on monocytes or macrophages, autoantibody burden or complement activation markers) (Ref. 89). Integration of these biomarkers with clinical features such as refractoriness to first-line therapies, bleeding phenotype and thrombosis risk may enable mechanism-informed patient selection for GPVI-targeted combination regimens.

Collectively, these considerations support a precision-oriented model in which GPVI-directed therapies are deployed selectively within combination regimens, matched to disease endotypes defined by platelet activation status and immune effector dominance (Refs. 72–74). Such an approach moves beyond empiric escalation of immunosuppression towards rational, pathway-guided modulation of pathogenic platelet–immune circuits in ITP.

### Biomarker development for GPVI-mediated disease activity

Effective translation of GPVI-targeted therapies into clinical practice hinges on the development of robust and actionable biomarkers that capture both platelet function and immune dysregulation (Refs 74, 90). Candidate biomarkers include surface GPVI receptor density, markers of platelet activation such as P-selectin expression or PAC-1 binding, formation of platelet–leucocyte aggregates and plasma levels of soluble GPVI or downstream inflammatory mediators (Ref. 91). These biomarkers provide mechanistic insight into the extent of GPVI engagement, platelet hyperactivity and thromboinflammatory burden in individual patients.

Dynamic, longitudinal monitoring of these indicators can serve multiple critical roles. First, they can guide dosing and scheduling of GPVI inhibitors to optimize therapeutic efficacy while minimizing bleeding risk (Ref. 12). Second, they can help predict which patients are most likely to respond, enabling early identification of non-responders or those at higher risk of adverse events. Third, functional biomarker readouts integrated with clinical endpoints – such as platelet counts, bleeding scores or immune activity – can inform adaptive trial designs, accelerating iterative learning and precision medicine approaches in ITP (Ref. 1).

Ultimately, biomarker-driven strategies facilitate a shift from reactive, platelet-count–centric management to proactive, mechanism-based therapy. By incorporating real-time assessments of GPVI activity and platelet–immune interactions, clinicians can tailor interventions to the pathogenic profile of each patient, maximize efficacy, reduce unnecessary exposure and refine the risk–benefit balance in GPVI-targeted ITP therapy.

### Current clinical status of GPVI inhibitors

Glenzocimab (ACT017), a humanized anti-GPVI Fab fragment developed by Acticor Biotech, has progressed into clinical testing for acute ischaemic stroke and related thromboinflammatory indications (Ref. 92). Early clinical data from the randomized ACTIMIS phase 1b/2a study indicate that glenzocimab (1000 mg IV) was well tolerated as an adjunct to alteplase and that treatment was associated with signals of reduced intracerebral haemorrhage and mortality in the studied cohorts, supporting further evaluation (Ref. 92). Follow-on adaptive phase 2/3 programmes have been registered and are actively evaluating glenzocimab in large acute-care populations (e.g. the ACTISAVE trial assessing glenzocimab as an add-on to standard of care in acute ischaemic stroke; NCT05070260) and additional trials (including GREEN and LIBERATE) are designed to assess efficacy in reperfusion and myocardial infarction settings (NCT05070260).

Importantly for ITP, current clinical development programmes for GPVI inhibitors are focused on acute thrombotic indications (stroke, reperfusion injury, myocardial infarction) and to our knowledge there are no registered clinical trials specifically evaluating GPVI inhibitors for immune thrombocytopenia at present (NCT05070260).

Consequently, translation of GPVI-targeted strategies into ITP will require dedicated early-phase clinical studies that (i) prospectively define mechanistic inclusion criteria (e.g. platelet activation signatures or GPVI expression/function), (ii) prioritize safety-focused endpoints including bleeding and immune competence and (iii) incorporate biomarker-guided adaptive designs to identify responsive patient sub-groups.

## Future directions

### GPVI modulation in immune thrombocytopenia: opportunities for precision medicine

Targeting GPVI offers a mechanistically focused complement to conventional, broadly-acting immunotherapies for ITP: pre-clinical and mechanistic human data indicate that GPVI participates in platelet activation and platelet–immune interfaces that can sustain immune-mediated platelet loss (Refs 26, 93, 94). However, clinical evidence of therapeutic efficacy in ITP is currently limited and GPVI-targeted agents have primarily been evaluated in thrombotic/ischaemic indications to date; therefore, any translation into ITP should be pursued within a precision-medicine framework that prospectively tests mechanism-based biomarkers.

Patient selection should be driven by mechanistic signatures (examples: elevated platelet activation markers, abundant platelet–leucocyte aggregates, low GPVI surface density or high soluble GPVI) measurable by standardized assays rather than platelet count alone; these readouts have been validated in translational studies of platelet immunobiology and PLA (platelet–leucocyte aggregate) measurement (Ref. 68). Trial designs should use these biomarkers as stratification variables or exploratory endpoints to identify sub-groups most likely to benefit and to limit bleeding risk by informing adaptive dosing.

### Innovative delivery systems: nanoparticles or platelet hitchhiking approaches

Several emerging delivery concepts could improve the therapeutic index of GPVI modulation, but each currently rests on pre-clinical proof of principle and requires focused translational work. For clarity: ‘platelet hitchhiking’ refers to ex vivo or in vivo strategies that load drugs or nanoparticles onto autologous platelets (or platelet membranes) so the platelet’s native trafficking concentrates payloads at sites of platelet activation; nanoparticles denote engineered carriers enabling controlled release and targeting; hydrogel encapsulation denotes biocompatible matrices that can locally retain and protect cells or drug carriers from immediate immune clearance (Refs 95–97).

Nanoparticles can be engineered to carry GPVI inhibitors, immune-modulatory agents or anti-inflammatory payloads with controlled-release kinetics, ensuring sustained local bioavailability while reducing peak-related toxicity. ex vivo functionalization of autologous platelets enables these cells to act as programmable carriers, homing directly to hyperactive platelet populations or microenvironments enriched in platelet–leucocyte aggregates (Refs 98, 99). These approaches are enabling technologies, not standalone clinical solutions at present. Early translational steps should therefore (a) prioritize safety profiling (immunogenicity, complement activation), (b) demonstrate that the platform preserves platelet haemostatic function and (c) show target engagement (e.g. local GPVI inhibition or reduction of PLA burden) in humanized or ex vivo human blood systems before first-in-human testing.

Hydrogel-based systems provide an additional layer of protection and control (Refs 100, 101). By encapsulating platelets or platelet-mimetic carriers within biocompatible hydrogels, it is possible to shield them from autoantibody-mediated destruction, complement activation and premature clearance, preserving both platelet count and functional competence. Hydrogels can be further functionalized with GPVI inhibitors, antioxidants or immunomodulators to create a synergistic, localized therapeutic microenvironment, reducing thromboinflammatory signalling while maintaining haemostatic capacity (Refs 102, 103).

These innovative delivery platforms also enable combinatorial therapeutic strategies. GPVI inhibitors delivered via nanoparticles, platelet hitchhiking or hydrogel matrices can be paired with TPO receptor agonists to simultaneously enhance platelet production and protect against immune-mediated clearance. Integration with systemic immunomodulators could further suppress inflammatory cascades and platelet–leucocyte aggregate formation, providing multi-level control over disease pathology.

Collectively, these advanced approaches – hitchhiking, nanoparticles and hydrogel encapsulation – offer a highly precise, patient-tailored platform to redefine GPVI-targeted therapy in ITP. By enhancing local efficacy, reducing systemic toxicity and enabling modular combination strategies, these technologies hold the potential to shift ITP management from reactive platelet support towards proactive, mechanism-driven, precision-guided interventions.

### Integrating GPVI inhibition into the ITP therapeutic landscape

Clinical development should emphasize combination and biomarker-driven adaptive trials. This approach is preferable to immediate monotherapy claims, which could overstate the current evidence base. A pragmatic roadmap includes: (1) early-phase studies in well-phenotyped patients with biomarker selection (e.g. elevated platelet–leucocyte aggregates or preserved haemostatic reserve); (2) primary safety and pharmacodynamic endpoints (bleeding scores, platelet function assays, GPVI receptor density, PLA frequency) and (3) exploratory efficacy endpoints (duration of platelet response, reduction in rescue therapy).

**Figure 2.**
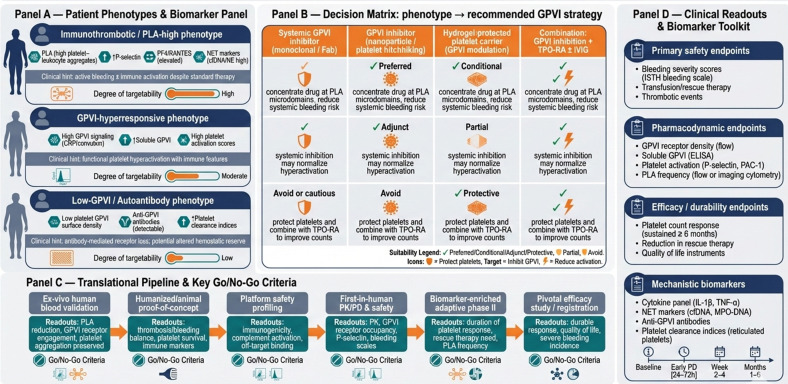
Conceptual translational roadmap for precision-guided GPVI-targeted therapy in immune thrombocytopenia (ITP). Panel (A) delineates mechanistically defined patient phenotypes based on platelet activation, platelet–leucocyte aggregates, GPVI surface expression and soluble GPVI levels. Panel (B) maps these phenotypes to tailored GPVI intervention strategies, including systemic inhibitors, nanoparticle or platelet-hitchhiked delivery, hydrogel-protected carriers and combination approaches with TPO receptor agonists or IVIG. Panel (C) outlines a staged pre-clinical-to-clinical pipeline with explicit go/no-go decision points and assay-based validation steps to ensure safety, target engagement and mechanistic efficacy. Panel (D) highlights prioritized clinical, pharmacodynamic and mechanistic readouts with recommended sampling intervals, including bleeding scores, platelet function assays, GPVI receptor density, PLA frequency and immune activation biomarkers. Together, this figure operationalizes biomarker-guided patient selection, innovative delivery platforms and adaptive trial design to translate mechanistic concepts into actionable, precision-driven ITP therapeutics.

A schematic figure (Figure 2) maps patient phenotype, biomarker panel, proposed technologies (GPVI inhibitor alone, delivered via platelet/nanoparticle/hydrogel carrier or combined with TPO-RA/IVIG) and anticipated clinical readouts (safety, platelet durability, reduced PLA). This figure highlights how mechanism-driven patient stratification, innovative delivery platforms and integrated trial design can operationalize GPVI-targeted precision therapy, bridging pre-clinical insights to actionable clinical interventions and establishing a roadmap for next-generation, individualized ITP management.

## Conclusion

### GPVI as a pathogenic driver and therapeutic node

Glycoprotein VI (GPVI) has emerged as a pivotal mediator in the pathophysiology of immune thrombocytopenia (ITP), integrating key processes that drive both platelet dysfunction and immune dysregulation. By orchestrating platelet hyperactivation, enhancing platelet–leucocyte aggregate formation and facilitating immune-mediated clearance, GPVI serves as a central nexus linking thrombosis, inflammation and autoimmunity. Clinical and pre-clinical evidence indicates that aberrant GPVI signalling contributes not only to quantitative thrombocytopenia but also to qualitative defects in platelet function, amplifying bleeding risk and sustaining pathogenic immune circuits.

Importantly, GPVI is predominantly expressed on platelets and megakaryocytes, distinguishing it from broadly expressed immune or haemostatic targets. This selective expression enables the design of therapies that specifically modulate pathological platelet activation without broadly compromising haemostasis. GPVI thus represents a dual-purpose therapeutic node: it is both a mechanistic driver of disease and a tractable point for intervention. Modulating GPVI signalling has the potential to interrupt the self-perpetuating cycle of platelet destruction and immune hyperactivation while preserving essential platelet functions critical for normal vascular integrity.

By positioning GPVI as both a culprit and a target, therapeutic strategies can shift from conventional immunosuppressive approaches towards precision-guided interventions. This framework allows for rational, patient-tailored treatments that focus on correcting underlying pathogenic mechanisms, rather than merely elevating platelet counts. Targeted GPVI modulation therefore offers the opportunity not only to alleviate symptomatic thrombocytopenia but also to re-shape the immune and thromboinflammatory landscape of ITP, opening avenues for durable disease control and improved clinical outcomes.

### Potential to transform ITP management from symptom control

Targeting GPVI holds the promise of fundamentally re-shaping the therapeutic landscape of ITP, moving care from reactive symptom management to proactive, mechanism-based disease modulation. By selectively inhibiting GPVI, therapies can simultaneously preserve platelet counts, enhance platelet functional competence and attenuate immune-mediated clearance, addressing both the quantitative and qualitative deficits that drive bleeding risk, thromboinflammatory complications and disease morbidity.

When combined with precision patient stratification, rational combination regimens (e.g. TPO receptor agonists, IVIG, corticosteroids) and biomarker-guided monitoring, GPVI-targeted interventions have the potential to deliver highly individualized, durable outcomes. This approach may reduce dependence on broad immunosuppressive therapies, minimize systemic side effects and improve overall patient quality of life.

Beyond individual patient benefit, GPVI modulation could re-define clinical decision making in haematology by providing actionable insights into disease activity, guiding therapy based on mechanistic drivers rather than platelet count alone. Ultimately, GPVI-targeted therapy exemplifies the integration of translational science into precision medicine, transforming ITP management from symptom-focused care into a proactive, targeted discipline with the potential for durable disease control, reduced morbidity and improved patient outcomes.

## Data Availability

This is a review study and it is not an original. Data availability is corresponding author responsibility.
